# An observational comparison of first-line combination antiretroviral treatment (cART) with 2NRTI and ATV/r or DRV/r in HIV-infected patients in Italy

**DOI:** 10.7448/IAS.17.4.19771

**Published:** 2014-11-02

**Authors:** Alessandro Cozzi-Lepri, Andrea Antinori, Stefano Bonora, Antonella Cingolani, Giovanni Cassola, Gioacchino Angarano, Vincenzo Vullo, Cristina Mussini, Andrea Gori, Franco Maggiolo, Antonella Castagna, Antonella d'Arminio Monforte on behalf of the ICONA Foundation Study

**Affiliations:** 1 Infection and Population Health, University College London, London, UK; 2 Infectious Diseases, INMI Spallanzani Hospital, Rome, Italy; 3 Infectious Diseases, University of Turin, Turin, Italy; 4 Faculty of Medicine, Cattolica Sacro Cuore University, Rome, Italy; 5 Infectious Diseases, Galliera Hospital, Genova, Italy; 6 Biomedical Science, University of Bari, Bari, Italy; 7 Public Health and Infectious Diseases, Sapienza University of Rome, Rome, Italy; 8 Infectious Diseases, University of Modena, Modena, Italy; 9 Infectious Diseases, San Gerardo University of Monza, Monza, Italy; 10 Infectious Diseases, Ospedali Riuniti Bergamo, Bergamo, Italy; 11 Infectious Diseases, San Raffaele Hospital, Milan, Italy; 12 Health Sciences, San Paolo Hospital, Milan, Italy; 13 Health Sciences, University of Milan, Milan, Italy

## Abstract

**Introduction:**

In a recent clinical trial (ACTG 5257), no difference in viral failure (VF) of a first-line cART containing atazanavir/r (ATV/r) or darunavir/r (DRV/r) was found [1]. For the endpoint of discontinuation due to intolerance, the regimen with DRV/r was superior to that of ATV/r (49% of the stops of ATV/r were attributed to jaundice or hyperbilirubinemia). These and other intolerances to ATV/r remain a concern for clinicians.

**Methods:**

Participants in the ICONA Foundation Study who started cART with 2NRTI+ ATV/r or DRV/r while ART-naïve were included. Several endpoints were evaluated: confirmed VF>200 copies/mL after six months of therapy, discontinuation of DRV/r or ATV/r for any reasons or because of intolerance/toxicity (as reported by the treating physician) and the combined endpoint of VF or stop. Survival analysis with Kaplan–Meier curves and Cox regression model stratified by clinical site was used. Patients' follow-up accrued from cART initiation to the date of the event or to the date of last available visit/viral load.

**Results:**

894 patients starting 2NRTI+ATV/r and 686 2NRTI+DRV/r when ART-naïve on average in 2011 (IQR: 2010–2012) were studied. Most common NRTIs used were FTC/TDF (84%) and ABC/3TC (12%). Median age was 40 years, 22% females, 44% heterosexuals. Patients starting ATV/r were more likely to be hepatitis B/C infected (2% and 14% vs 1% and 9%, p=0.001), they started one year earlier (2011 vs 2012, p=0.001), were more likely to be enrolled in sites located in the north of Italy (63% vs 54%, p=0.04), started cART less promptly after HIV diagnosis (5 vs 2 months, p=0.02) and less likely to have started TDF/FTC (83% vs 85%, p=0.02). By two years of cART, 9.8% (95% CI 7.6–12.0) of those starting ATV/r experienced discontinuation due to intolerance/toxicity vs 6.5% in DRV/r group (95% CI 4.2–8.8, p=0.04). After controlling for several potential confounders (age, gender, nation of birth, mode of HIV transmission, hepatitis co-infection status, AIDS diagnosis, nucleoside pair started, baseline CD4 count and viral load and year of starting cART) the relative hazard (RH) for ATV/r vs DRV/r was 2.01 (95% CI 1.23, 3.28, p=0.005). There were no statistical differences detected for any of the other outcomes.

**Conclusions:**

Although unmeasured confounding cannot be ruled out, our results seem to be consistent with those of the ACTG 5257. When all cause discontinuations were considered, or the composite endpoint of treatment failure, there was no difference between ATV/r- and DRV/r-based regimens.

**Table 1:** Relative hazards of reaching the various defined outcomes from fitting a Cox regression model

Outcomes	Unadjusted RH (95% CI)	p	Adjusted RH (95% CI)	p
All cause discontinuation				
ATV/r vs. DRV/r	1.18 (0.97, 1.43)	0.09	1.16 (0.92, 1.47)	0.20
Discontinuation due to toxicity				
ATV/r vs. DRV/r	1.48 (0.98, 2.22)	0.06	2.01 (1.23, 3.28)	0.005
VF>200 copies/mL				
ATV/r vs. DRV/r	3.49 (1.89, 6.43)	<.001	1.63 (0.75, 3.54)	0.22
VF>200 copies/mL or discontinuation				
ATV/r vs. DRV/r	1.25 (1.02, 1.52)	0.03	1.14 (0.90, 1.45)	0.27
